# Laboratory Practices, Potentiality, and Material Patienthood in Genomic Cancer Medicine

**DOI:** 10.1177/01622439231172571

**Published:** 2023-05-04

**Authors:** Julia Swallow, Tineke Broer, Anne Kerr, Sarah Cunningham-Burley

**Affiliations:** 1 Usher Institute of Population Health Sciences and Informatics, Old Medical School, The University of Edinburgh, United Kingdom; 2 Tilburg University, Noord-Brabant, the Netherlands; 3 University of Glasgow, United Kingdom; 4 University of Edinburgh, Lothian, United Kingdom

**Keywords:** genomic medicine, laboratory work, oncology, patienthood, potentiality

## Abstract

Laboratory practitioners working in oncology are increasingly involved in implementing genomic medicine, operating at the intersection of the laboratory and the clinic. This includes molecular diagnostic work and molecular testing to direct entry into molecular-based clinical trials and treatment decision-making based on molecular profiling. In this article, we draw on qualitative interviews with laboratory practitioners in the United Kingdom to explore the role of laboratory work in genomic cancer medicine, focusing on the handling of patient tissue and making of potentiality to guide patients’ present and future care. With an increase in molecular testing to inform standard care and clinical trial participation, we show how practitioners “potentialized” the tissue by carefully negotiating what to test, how to test, and when. This included maximizing and managing small amounts of tissue in anticipation of possible future patient care. Tissue archives also took on new meaning, and potentiality, which practitioners negotiated alongside patient care. Potentiality was key to generating the “big” future of genomic medicine and also involved care work where the tissue emerged as an extension of the patient, as a form of “material patienthood,” to secure present and future care for patients through their involvement in genomic medicine.

## Background

Through advances in cancer genomics, molecular techniques are being introduced in the laboratory in order to subtype cancers and provide more accurate molecular information (or predictive biomarkers) to guide diagnosis and treatment decision-making. This molecular work occurs alongside traditional morphological work, such as looking at a sample to determine if it contains a tumor, and whether the growth is benign or malign (Domagala-Kulawik 2019). Genomic medicine as discussed in social science literature is often heralded as an entirely new way of practicing medicine, for instance, by blurring the boundaries (see Star 2010) between research and care or between bench and bedside (Cambrosio et al. 2018). Yet only few studies emphasize the role of laboratory practitioners in conducting genomic medicine (see Bourret, Keating, and Cambrosio 2011), which as we show involves negotiating new forms of genomic knowledge, and also keeping the patient in mind and advocating for their best interests despite not treating patients directly.

Tissue is crucial to the aims and practices of genomic medicine, so in this article, we focus on laboratory practitioners to explore how they work with the tissue at the center of genomic medicine (see also Bogicevic et al. 2020; Swallow et al. 2020). Bogicevic et al. (2020, 185) adopt the term “somatic mode” to conceptualize how tumor tissue in genomic medicine “comes to be enacted as detached from the person—as a biological resource concerning the present by informing possible treatment actions in an individual.” Swallow et al. (2020) also discuss tissue as a key actant in the accomplishment of genomic medicine, where an increasing number of tests are carried out on tissue, and careful negotiation is required to establish what to sample, where to sample, and how much to sample to ensure quality and quantity DNA to direct patient care, both now and in the future.

We build on this work to explore how laboratory practitioners worked to potentialize (Taussig, Hoeyer, and Helmreich 2013) the tissue that is undergoing molecular analysis. We draw on Science and Technology Studies (STS) and anthropological literatures on the interconnected practices of potentiality, vitality, materiality, and care to theorize how the tissue connected practitioners to a patient’s present and future care. In so doing, we argue that tumor tissue is a conduit for both future-looking practices *and* present-oriented practices for patients, where the tissue is crafted as a form of material patienthood rather than detached from the patient (cf. Bogicevic et al. 2020). Theorizing and rendering visible potentializing practices is crucial for understanding the making of “big” genomic medicine (see Michael 2017; Kerr et al. 2021) and “small” individual patient futures alike.

## Theoretical Inspirations: Potentiality, Materiality, and Care

Our article builds on literature in the sociology and anthropology of contemporary biomedicine that discusses anticipation, expectations, potentiality, and temporality across healthcare and research. The sociology of expectations, which attends to how promissory visions and expectations of technological innovation drive research and development in the present (Borup et al. 2006; Van Lente and Rip 2012), invites us to explore how expectations are performative and closely tied to future-making. Literature on the sociology of the future also draws our attention to notions of possibility or potentiality, which are key to these processes. As Adams et al. (2009, 249) argue, the future is always uncertain and at the same time is inevitably on its way and therefore “always demanding a response.” Thus, the “management of the future becomes a pre-occupation of the present through the obligatory passage-point of “possibility” (Adams et al. 2009, 259).

Sociology and STS literature on promise and expectations critically interrogate speculation, anticipation, and temporality, paying due care not to present futures “in definite and knowable terms” (Taussig, Hoeyer, and Helmreich 2013, S10). Decentering futures in their analysis of contemporary biomedicine, they focus on potentiality and how this “retain[s] a larger degree of ambiguity” (Taussig, Hoeyer, and Helmreich 2013, S10): in other words, potentiality is future-oriented but does not presuppose that the future is knowable.

In biomedical practices, potentiality indexes a gap between what is and what might, could, or even should be. Such a gap opens up an imaginative space of magic and mystery in which future-building activities related to animating bodies and extending life in new ways loom large. (Taussig, Hoeyer, and Helmreich 2013, S5)

Potentiality is both an analytical category and an empirical object of study—an approach we adopt in this article to explore how genomic medicine is future-oriented *and* present focused (Bogicevic et al. 2020). Expectations and potentiality are, however, not just discursive but deeply material practices (see, e.g., Landecker 2009; Waldby 2019). By ensuring a future for the tissue, laboratory practitioners imagine and simultaneously craft a potential future life for a patient. We call this material patienthood to capture the presence of the patient in the material practices adopted in the laboratory, now and for the future.

Although the term “material patienthood” is our own, our definition and analysis borrows from other STS and anthropological work on biomedical materiality, vitality, and potentiality. For instance, we draw from STS and anthropological scholars who have explored how biological matter potentiates (Waldby 2019) or gains value (biovalue) through specific cultural and technological processes (Mitchell and Waldby 2006; Landecker 2009; Waldby 2019). This invites us to consider how tissues can take on symbolic value as precious be that because of their life-affirming potential, for example, for participants considering embryo donation to stem cell research (Parry 2006) or in relation to media and public concern about embryonic stem cells (Williams, Kitzinger, and Henderson 2003; Doring and Zinken 2005; Kitzinger and Williams 2005). We must consider how potentiality changes over time: material changes in the tissue as well as changes in the techniques to “read” the tissue in new ways. Bogicevic et al. (2020) reflect on how the materiality of tumor tissue changes, related to what Lappé and Landecker (2015) describe as “genetic instability.” In order to drive patient care (treatment options), it is critical to have information about the “present state in the tumor,” which is difficult because cancer is always on the move (Bogicevic et al. 2020, 187). In addition to the changing nature of tumor mutations, practitioners also have to be mindful of as-yet-unknown changes in knowledge, healthcare practices, and indeed laboratory practices themselves, as they continually potentiate tissue’s value. The tissue, then, is an object of care itself to care for the present and future of patients as individuals and as a collective.

At the same time, we must consider how biological material is produced and tended to in order to retain or develop its potential. Lee (2016) contributes to the literature on tissue economies to examine how tissues, including placentas, are produced through complex relations of care, which she calls economies of care. Lee (2016, 459) refers to care in this context as “a set of practices through which ‘life’ [or vitality] is maintained and continued,” and potentialized (see also Svendsen 2011), noting that “tissues require care from many different bodies to ‘realize’ what is assumed to be their vital potential.” Laboratory workers “take care of cells in anticipation of something good that will unfold from their cellular vitality” (Lee 2016, 468). Friese (2013) also argues in her work on model organisms in translation medicine that care potentializes^1^:

Care is a potentializing practice. Care is central to the everyday idea of potential itself. In its most common valence, potential denotes the idea that someone or something must be nurtured so that a kernel of ability or talent is actualized in practice. (Friese 2013, S130)

Potentiality, vitality, and care are thus closely connected, where working to potentiate often means caring for, for example, a patient (or life, in Lee’s words), and crafting or extending a particular future for them. This crafting can be done discursively and also, as we will show, materially.

In this article, we explore how laboratory practitioners potentialized the tissue to extend or provide a present and a more distant future for cancer patients through practices of care across time—through involvement in a clinical trial and/or to guide the use of targeted therapies now or in the future. Extending the idea that potentiality is always concerned with future-oriented practices, we argue that potentializing the tissue involved navigating co-existing temporalities: negotiating practitioners’ concerns about patients’ present and immediate care (Bogicevic et al. 2020), and at the same time crafting a potential, molecular future. Care for the tissue also connected practitioners to patients because they handled patient tissue in the laboratory and was driven by hope for anticipated futures (further treatment, future trial involvement) that may (or may not) arrive (Adams et al. 2009). Despite generally being, at least physically “at a distance” from patients, laboratory practices configured new forms of material patienthood through the tissue, with practitioners (re)imagining patients in their work, bringing them to the fore in their everyday practices.

## Method

The data on which this article is based is drawn from a large multisited ethnographic research project examining how genomic medicine in cancer is impacting patient and practitioner perspectives and experiences of cancer research and care (Translations and Transformations in Patienthood: Cancer in the Post-genomics Era). The research project was approved by the relevant National Health Service (NHS) Research Ethics Committee (REC number: 16/YH/0229). This particular article draws on semistructured qualitative interviews carried out with laboratory practitioners involved in pathology work in oncology across NHS and university sites in England and Scotland.

Across the research sites, we carried out sixteen interviews with laboratory-based practitioners working across a range of cancer types. All respondents were involved in handling, processing, sampling, analyzing, and interpreting solid tissue or blood samples for both routine morphological diagnostic work and for molecular work. Practitioners included pathologists (both histopathologists and hematopathologists), biomedical scientists, geneticists, and clinical scientists. Their specific roles in the application of genomic cancer medicine is as follows. Pathologists, including those working at the level of consultant, were involved in tumor classification to direct diagnosis and prognosis as well as treatment decision-making based on molecular profiling, with several consultants attending weekly multi-disciplinary team (MDT) meetings. Biomedical scientists supported the pathology service and were involved in preparing and analyzing solid tissue or blood samples, extracting and analyzing DNA from samples for both standard care and research, and supporting diagnostic pathways. Alongside their work in assessing patients’ risk of cancer (identifying germline mutations), geneticists worked with tumor tissue to identify genetic variants within the tumor (somatic variants), information used to guide treatment decision-making. Clinical scientists worked alongside pathologists, biomedical scientists, and geneticists using molecular techniques and technologies to process tissue and blood samples, and analyze and interpret genomic results for research and care.

Interviews were semi-structured and carried out by members of the research team between 2016 and 2019 and lasted approximately one to one-and-a-half hours, and we asked questions about how practitioners fitted molecular work into existing practice, the challenges and opportunities associated with molecular work, with an emphasis on how the tissue was handled. Interviews were audio recorded and transcribed verbatim. We adopted a situational analysis approach to analyze interview transcripts thematically, and we dealt with data manually (see Clarke, Friese, and Washburn 2016). Key themes were developed with the research team and included potentiality, care, value, uncertainty, futures, and expectations.

We move on to describe how laboratory practitioners worked to potentialize tissue as part of their everyday activities directed at securing patients’ present and future care. We begin by showing how potentializing the tissue to direct present and future patient care involved negotiating small tissue samples to maximize the tissue for both diagnosis and molecular testing. We then show how lab workers potentialized tissue by working to ensure quality samples, which included managing the unpredictability and precarity of tumor material (both preserved tissue and in vivo tissue) as it changed over time, also keeping the patient in mind when making decisions about accessing better quality tissue—accounting for the “biographical life” of the tissue (Svendsen 2011). In the final section of the analysis, we consider laboratory archives as a means through which tissue’s potentiality could be maintained and secured for patient care in the distant future. Across the analysis, we focus on the “future-building activities” (Taussig, Hoeyer, and Helmreich 2013, S5) at the center of pathology work and argue that pathology work configured new forms of material patienthood through the tissue in the laboratory.

## Findings

### Maximizing the Tissue

In this first section, we analyze how laboratory practitioners navigated the challenges posed by conducting molecular medicine, paying particular attention to the use of tissue. This included working with small amounts of tissue which was increasingly needed for both routine diagnostic and molecular work for research and clinical purposes. Working with small amounts of tissue could be a result of difficulties accessing and obtaining tissue, particularly in areas such as nonsmall cell lung cancer. Patients are commonly diagnosed with nonsmall cell lung cancer when the disease has metastasized and only a small number of patients undergo curative surgery where large tissue samples can be obtained through open-chest surgery. Patients whose disease has metastasized often undergo needle lung biopsies to collect cell samples, a procedure that can be risky for patients due to the possibility of it causing a lung collapse (Hiley 2016). These procedures may also not yield a sample of sufficient quality or quantity to carry out both diagnostic and molecular testing (Hiley 2016). It may also be difficult to carry out repeat biopsies for the purposes of molecular testing if patients are significantly unwell, given the risks associated with both surgery and needle biopsies. In areas such as breast cancer, surgery to remove the tissue is more common, and larger quantities of tissue can be obtained, particularly if the disease has not metastasized. Yet difficulties remain concerning increased testing being carried out on all tissue samples for both diagnostic and molecular purposes, as we show below.

A range of laboratory practitioners discussed the challenges associated with getting a large enough sample and, second, getting enough cancer tissue in the sample as illustrated in the extract below:

Lung samples tend to be quite small biopsies, like, very, very tiny, less than half a centimeter, which means that then you’ve got problems with how much tissue you can use, you’re not going to get very much DNA out of a very small biopsy and the pathologist may have already cut several sections in order to do the diagnosis. So, sample size is a problem, particularly for lung cancer. (Pathologist 6)

The size of the tissue for analysis differed depending on the research study or trial and specific type of cancer. During interviews we were often shown tissue samples, and laboratory practitioners frequently commented on the quality of the biopsy by talking about “a good core [sample]” or “a generous biopsy,” for example. Many of the respondents suggested that the increase in molecular testing in laboratories has led to an influx of additional tests on limited tissue samples, which could be challenging to negotiate:

Number of challenges. Within pathology we’ve had a lot of—well we’ve seen a lot of increased workload over recent years. That’s partly been addressed by an increase in the number of pathologists to deliver that increased workload, but we see a lot more specimens per year. We’re expected to do a lot more with the specimens now. (Pathologist 2)

As Pathologist 2 explains, the increase in molecular testing has led to a significant increase in workload; fitting the molecular work into existing diagnostic and histological work was described by Pathologist 1 as a “sea change.” In addition to the “traditional” diagnosis of cancer and where the cancer originated, increasingly specimens are tested for particular subtypes of, for instance, lung cancer, which might change both the patient’s outlook and treatment. However, the quantity of tissue with which practitioners are expected to work with does not change despite changing expectations about how the tissue should and might be used, and its potential utility in the future, as Pathologist 2 goes on to reflect:

Like a little millimeter of tissue so you’re working with not very much material than that. So, there are a number of major problems, when it comes to finding adequate material to test…we generally now test the resection [surgery to remove tissue/a tumor] because it’s got more material in it.

Seeking out adequate material in an effort to potentialize and secure the tissue as future resource also required practitioners to act with care and caution when deciding which tests to carry out:

It’s a challenge for us as individual pathologists because we constantly need to be thinking: what else might we want to do with this tissue, we’ve not got very much of it, okay, so rather than having the luxury of thinking “oh I wonder if it could be that?” Or “I wonder if it could be this one [this sub-type/mutation]?” You know “oh I don’t think it is but maybe we should just make sure that it’s not that” you know, you have to be constantly thinking “do I really need to do that, do I really need to do that because that’s going to use tissue and I may need that tissue for something else down the line?” (Pathologist 4)

The possibility that the tissue may be of use at an (uncertain) point in the future was difficult for many of the practitioners to manage; potentializing the tissue meant protecting and preserving the small quantity of tissue, to be sure of a diagnosis before molecular testing, and to constantly check their own practice, “do I really need to do that?” Efforts to maximize or potentiate the vital tissue was a predominant theme across interviewees’ accounts, described by Pathologist 6 as “trying to make the best use of material” which in areas such as lung cancer meant conserving tissue in anticipation of a potential molecular future. As they further explained, “we do our Hs and Es,^2^ we do our immune and we stop because these are small biopsies and we don’t want to, you know, do too much because molecular could be needed.” In order to “manage the anticipated” (Adams et al. 2009), practitioners worked to make the best use of tissue and maximize its potential, as Hematopathologist 1 confirmed: “people are thinking more about the tissue and the pathology and maximizing the benefit from it…it becomes very important how we handle the tissue and it’s going to be more and more important.”

Navigating the context in which the tissue would be used also meant keeping the patient in mind when requesting samples as part of a wider and more coordinated MDT approach as Histopathologist 1 went on to explain,

That’s the beauty of MDT, we made a decision [about the desired/potential treatment], and so therefore I was able to then only ask and request for that particular test and that particular tissue. And that’s how we save the tissue. So that’s being sensible and clinically—using your clinical judgment to sort of decide, you know, what is appropriate. And again, not me on my own but the group.

Many of the laboratory practitioners discussed being very careful in what they will and will not do with tissue—only when the patient is likely to benefit from a certain molecular test would they suggest it. As Biomedical Scientist 1 explains, “we just try and make sure that we make best use of the tissue.” The potentiality of the tissue was therefore tied to both efforts to secure patients’ possible (molecular) futures, and navigating what would be immediately beneficial for patients in the present.

Practitioners were concerned to use tissue economically and wisely—concerns that were emphasized in the context of new and possible future molecular tests, which in the short and long term could guide treatment options, including targeted molecular-based therapies when a patient’s cancer stops responding to treatment.^3^ In anticipation of future molecular testing, practitioners had to carefully balance doing enough tests to make a firm diagnosis (because the wrong diagnosis can do a lot of damage: “it makes life very hard” for patients, as Pathologist 6 remarked), and on the other hand, minimizing the number of tests so the tissue is not wasted or ruined for future analysis. This anticipation of, and care for, a future for patients, despite not knowing what/when this might be, drove action in the present and efforts to maximize the tissue as a key actant in patient care (Swallow et al. 2020). Laboratory practitioners were therefore thinking ahead to what might be needed of the tissue, keeping the molecular in mind and attending to its biographical life (Svendsen 2011) by maximizing small samples and minimizing the number of tests.

### Managing (Potential) Changes in the Material and Its Use

Alongside negotiating small tissue samples, tissue must also be handled to ensure quality samples that accurately reflect the tumor status and progression. In particular, potentializing the tissue was linked to the need to contain or manage unpredictability and precarity of samples and vital tissue, which changes over time. A tissue sample captures a moment in time, and because DNA degrades over time, there are limitations on how the tissue may be used in the future (see Bogicevic et al. 2020). In terms of vital, in vivo tissue, cancer is dynamic and continually changing, always “on the move.” Tissue is lively and vibrant (Bennett 2010), for instance, in relation to biomarkers and genetic information, which makes securing a future complex. As Clinician Scientist 1 explains,

Cancers evolve very rapidly, cells divide very quickly, and genetic changes happen all the time, it’s a bit like evolution of man. And those that make the tumor cells more efficient grow faster. So, it—resistance [against a treatment] develops very quickly in the vast majority of people with BRAF inhibitors [drugs targeting the BRAF protein that might play a role in some tumors, including some melanomas].

Cancer’s continually evolving nature presents challenges to laboratory work, as it does in patient care. Patients’ tumors develop resistance to treatments and this means having to make difficult decisions about rebiopsying to test new mutations and to direct future treatment. Pathologist 6 discusses each of these challenges:

Certainly most of the solid tumors we deal with very quickly develop resistance to most, in the vast majority of patients, they develop resistance quite quickly…[for instance] where the initial drug would be active against EDFL mutations in lung cancer and in 50 percent of the people who develop resistance to that it’s because they’ve developed a second mutation and Tagrisso [a new drug] is active against the second mutation. So, then you need to test the second mutation, which is great news for molecular pathology laboratories ‘cause it keeps you occupied but it does mean that they need to have some kind of second sample which isn’t always that easy to achieve. Depending on what kind of tumor the person has, lung cancer is a terrible one for trying to get samples out of people at all, so you need some kind of method of taking a second sample ‘cause testing the original that you’ve got in your archives isn’t appropriate.

As Pathologist 6 notes, this impacted whether to test archived tissue, arguing that this may be futile because the identity and makeup of the cancer is changing continuously. Potentializing the tissue meant containing its precarity and handling tissue in a timely manner, but it also meant keeping the patient in mind when making decisions about obtaining new tissue for analysis through an additional biopsy. Given the risks associated with such a procedure, rebiopsying patients to obtain new tissue had to be carefully balanced in anticipation of molecular testing in the (distant) future, as Geneticist 1 explains:

I suppose in terms of repeat biopsies it’s, I guess, a bit more difficult with that because quite often patients can only come in once and sometimes they’re too ill to have repeat biopsies. So, I think that’s something that probably will evolve a bit more and there probably will be more discussion about that. But that really depends…[whether] the molecular test is going to be [a] crucial factor for what happens next with patients [whether and how to treat them]. You know you can’t really get somebody to go in for a repeat biopsy just to get a molecular result that might not mean anything, so….

Geneticist 1 keeps both the patient and their potential molecular future in mind when making decisions concerning rebiopsying (Adams et al. 2009; Friese 2013). Maximizing the tissue to realize what is assumed to be its vital potential (Lee 2016) had to be balanced alongside the need to protect patients from harmful procedures in the present (see Bogicevic et al. 2020) and uncertain (molecular) futures.

Such future-building activities related to efforts to potentialize the tissue (i.e., to secure quality samples) differed depending on the context in which the tissue was being used (i.e., diagnostic or research purposes). As Pathologist 4 explained,

It’s different if you failed to make a diagnosis because then you’ve got more of a justification for doing another procedure, but if you’ve got enough to make the diagnosis but you’ve just not got enough to do the molecular testing, then it’s a more finely balanced decision.

This tension between research and care was reflected on by Histopathologist 1:

But in most cases the prime objective is to look for a diagnosis…you need to get that diagnosis sorted first, and then go on to the bits and pieces. So, when I talk about good quality tissue, good quality tissue is really defined in the context of what you are trying to do. If you say this is poor quality, it is poor quality for doing sequencing work, but in majority, 99.9 percent of the cases, treatment decisions are made on histological assessment. So, is it bad quality tissue? No, it’s very good quality tissue, it’s very good tissue for the patient’s pathway….

So, as far as I’m concerned, as of now it’s good quality tissue that they’re getting. It’s not good quality tissue for the research projects which includes SMP [Stratified Medicine Programme] and Genomics, the Genomes programme. Because their expectation of what they want to get from the tissue is different to what the patients require. So that is the kind of thing…the diagnosis and patient management comes first, and the research comes second.

Here, we see the ways in which expectations of use attached to the tissue (see Svendsen 2011) differed for research and patient (diagnostic) care, noting also that these boundaries are not so clear cut (see Cambrosio et al. 2018). The “big” or promissory expectations of tissue which were tied to the future of genomic medicine through studies such as the UK-based 100,000 Genomes Project differed from the on-the-ground, here and now, expectations of tissue and its potential for immediate patient care. Potentiality opens up a space where visions for the future of cancer medicine “loom large” (Taussig, Hoeyer, and Helmreich 2013, S5), which sits alongside the on-the-ground expectations of tissue and its potential for present patient care, including both diagnosis and treatment. Realizing the “vital potential” of tissue involved negotiating expectations of value, which shifted depending on what was immediately valuable to patients (Svendsen 2011; Bogicevic et al. 2020) and thus required keeping the patient in mind as someone in need of care.

Practitioners worked to contain tissue’s unpredictability as it changed over time, as the disease developed in the patient; they also balanced the need to ensure a quality sample alongside the need to protect patients from harmful procedures in the present and from uncertain (molecular) futures. In so doing, laboratory practitioners continually kept patients in mind through the tissue that they were handling. We see here that the tissue was not completely detached from the patient (cf. Bogicevic et al. 2020), and this constructed its potentiality both in terms of directing present care (treatment or trial entry) and future (molecular) care and research. At times, this meant balancing the tensions between present care and (future) research, where care, and the patient needing care, always had to come first.

### Maintaining Potentiality by Storing the Tissue

We close our findings by showing that potentializing also involved accumulating tissue for archiving, maintaining its potential as a resource for future use (Lee 2016). This opens up a future for patients, seen here with respect to the possibility of generating molecular futures for individual and collectives of patients.

Tissue, including in the form of paraffin blocks, is stored in hospital pathology archives as routine practice.^3,4^ Going back to patient tissue for treatment reasons was not previously part of routine practice, so the archive of paraffin blocks “is now becoming more and more useful and relevant for patient management,” as one pathologist reflected. Here, we analyze a different temporal orientation to that discussed in the previous section—the distant future “potentiality” (Ganchoff 2004; Franklin 2006) of the tissue stored in archives. Our respondents indicate that the value of this archive is changing due to new molecular understandings, techniques, and treatments. Indeed, the tissue stored in archives is (increasingly) (re)analyzed in the context of genomic medicine; a means of maintaining tissue’s potential for distant future care, emblematic of the “big futures” associated with genomic medicine (see Michael 2017).

When asked during interview whether the paraffin block will remain part of clinical practice, one pathologist explained that in some cases, it is useful to return to archived material:

I think the block will [remain] because that’s your archived tissue, okay, I mean, that one, in the box there is a case from the 1990s…and the reason it’s out is this patient had a colon cancer resected in the 90s, these are the blocks from there, they then came back [years later]…with a lump on their lung, which got chopped out and was thought to be a metastasis from that. But the surgeon phoned me up the other week there and said, “they’re still alive, are you sure this wasn’t an early colon cancer and an early primary lung cancer, both of which have been cured, and can you have a look at it again?” So, we did and it looks like metastatic colon cancer, so they’ve just been lucky, they’ve had a genuine isolated single metastasis. (Pathologist 4)

A number of practitioners we spoke to reflected on the difficult balance between sequencing patients for whom their cancer has metastasized or testing “older,” archived samples. This pathologist, for instance, talked about the possibility of testing older samples from patients’ deceased relatives when patients are wanting to know if they have Lynch syndrome, a genetic condition predisposing people to colon cancer and other cancers:

If the patient has one of those mutations, I don’t know what the tipping point is between cost effectiveness for either sequencing all of those genes for the patient that’s sitting in front of you, or for us dragging stuff out from 1985 and trying to test something that’s really old. They don’t tend to work as well. They were never fixed as well. So, I don’t, there’s a tipping point in there that you think at some point this becomes ridiculous when we’ve got people scurrying around in basements trying to find a block from God knows when. (Pathologist 5)

Testing older samples requires additional labor in the form of working through the archives to test old material where there is a great deal of uncertainty about the vitality of the tissue and its potential use, given the likelihood of DNA degradation. By contrast, other practitioners remarked that accumulating tissue for potential future use is critical to driving forward molecular profiling and for directing patients’ care. Pathologist 3 explains why it is crucial to save the tissue:

So say if this same patient comes back and they have…if they had a cancer, and they get a lump and the lump doesn’t look like what was reported in the written report, the clinician can say “can you review this case that we’d sent you a biopsy in 2003, ‘cause I think either something funny has happened or there was a misdiagnosis or the patient has a new tumor.” So we get the block out of the file, we get the slides out the file, we look at the slides again. If you want to take more sections off the block and do some new antibody stains which we can do now which weren’t available in 2003, we can do a whole series of new stains now and we can say “yes, the diagnosis was correct and we agree with it” or “no, with the new ways we have of analyzing the tissue now we would write a slightly different report and call it something slightly different,” or say “yeah that was all correct and the thing the patient has now is something new and different.”

…the thing is you don’t know which ones you’re going to have to go back to at the time, so you have to save them all, because when you do have to go back it’s extremely useful, it’s really very valuable. But you don’t know which ones you’ll go back to.

Interviewer: Do you think with the advance of genomic techniques you will go back to more of those tissues?

We are already, yeah.

Accumulating and archiving tissue by adopting a “just in case” approach contributes to the wider knowledge economy of biomedical innovation, as Pathologist 4 explains:

So we will, from an R&D point of view, have a lot more data on the molecular signature, if you like, of our lung cancer patients than will be available for, for treatment at this stage, but at least it means there’s a bank of data there that when somebody comes along with a new drug for, I don’t know, MET amplification or PI3 kinase or something like that, we can say well we know that X percent of our patients have this mutation and therefore might be suitable for that. So, [the archive] informs, it’s part of the sort of academic background if you like, it informs the…the knowledge base I suppose.

In practice, however, practitioners involved in handling tissue discussed not wanting to waste resources or do unnecessary tests on tissue in a financially constrained NHS system. Although a bank of data might be useful to contribute to the “knowledge base” of cancer, this had to be carefully negotiated, given the need to conserve resources and space and ensure that it is not the pharmaceutical companies that, through access to data gathered in clinical trials, “run away with the family silver,” as Pathologist 4 explained.

Archiving tissue emerged as a means to maintain the potential of the tissue in anticipation of future individual patient care and to contribute to the wider knowledge economy by building a “bank of data.” Future-building activities take the form of pathologists adopting a “just in case” approach (Taussig, Hoeyer, and Helmreich 2013), archiving samples despite this (distant) future remaining uncertain. Archiving or maintaining potential was closely related to care for the future bioeconomy and knowledge economy of cancer medicine as well as for (and interlinked with) individual patients and their descendants (Mitchell and Waldby 2006; Friese 2013).

## Discussion

Across this article, we have analyzed the role of laboratory work in genomic cancer medicine where laboratory practitioners, as part of their everyday practice, worked to potentialize tumor tissue in response to challenges associated with the introduction of molecular medicine in cancer pathology. It included working with small tissue samples, negotiating the need for new biopsy tissue when the cancer changes while reducing harm to the patient and ensuring a quality sample for future molecular work. We demonstrated potentializing as a series of future-building activities via the tissue, carefully balanced with the need to protect patients here and now, for example, by making difficult decisions about accessing new and quality tissue, conserving tissue, and not wasting tissue. Our findings complement Bogicevic et al.’s (2020) emphasis on the present arrangements of genomic medicine (the “somatic mode”), drawing attention to how securing tissue potentiality was a process of navigating and anticipating an uncertain (molecular) future alongside present patient care. In this way, potentiality was both future-oriented and present-oriented. Focusing on the everyday work of potentializing, we also decentered analysis of promissory or “big futures” of genomic medicine (Michael 2017) which dominate social science literature. Instead we paid attention to the crafting of patient futures as part of everyday practice (Kerr et al. 2021), with a focus on the role of laboratory practitioners. Practices of potentializing were entwined with efforts to secure or extend patient futures in the context of wider promissory hopes or expectations attached to genomic medicine (like archives that may increase in potentiality with improved knowledge and techniques), which might mean going back to already archived tissue and building archives for future use. Like Lee (2016) and the sociology of expectations more generally, we do not see the tissue as having intrinsic potential and instead acknowledge that its potential was crafted, negotiated, and realized in relation to the making of patients’ present, near future, and distant (molecular) future care.

In social science literature on genomic medicine, the role of laboratory practitioners is not often discussed in depth. Yet we have shown their role to be crucial and of interest for social scientific analysis, because they elaborate notions of potentiality and future making in their everyday work. We have described practitioners’ (re)imagining and caring for patients by caring for the tissue sample. In this way, practitioners were working with material patienthood, treating the tissue as an extension of the patient, and in this way keeping the patient in mind. Yet, a potential future set of genomic treatments for patients (however uncertain this may be) had to be carefully balanced with patients’ care needs in the present or near future. This could include molecular profiling of existing tissue but also weighing up the value of further biopsies. Caring for the patient in the present involved considering their (uncertain) future and, as such, is a potentializing practice (Friese 2013) connected to securing a potential future for patients through molecular-based treatments and entry into clinical trials, while also carefully managing the risks associated with additional biopsies or the likelihood of successful treatment balanced against risks and harms.

Analyzing laboratory practitioners’ work we also begin to see the making of different kinds of temporalities which included present, near distant, and distant future care for individual cancer patients as well as patients in the collective through efforts to secure the wider knowledge economy of genomic medicine. The emergence of coexisting temporalities links closely both to the notion of potentiality and to the sociology of expectations, where potentiality and expectation practices, discursively and materially, coconstitute the future they refer to. We have shown that potentializing the tissue involved navigating care and competing expectations as part of future-building activities. At times, this meant not undertaking molecular analysis, which is to say that potentializing was not necessarily a means of performing promissory expectations, futures, or hopes of genomic medicine which “loom large” (Taussig, Hoeyer, and Helmreich 2013, S5). To care for patients could mean prioritizing everyday and ongoing patient care in the present, with a keen eye for the risks and uncertainties associated with more or less testing. In other words, the potentiality of tissue for (future) molecular analysis was not always prioritized in the UK laboratories we studied. This means that focusing on potentiality is inherently care work. Rendering such potentializing practice visible and addressing the coexistence of present and future-oriented activities is critical to wider discussions of everyday practice in genomic medicine. As genomic medicine develops, tissue continues to connect laboratory practitioners to patients in a complex landscape of research and care, and commercial profit. The materiality of the tumor tissue is not detached from the biographical life of the patient, and this constructs its potentiality by directing present treatment and also possible future (molecular) care.

## Notes

1. For further analysis of care as a potentializing practice, see Svendsen (2011), Svendsen and Koch (2013), and d’Hoop (2021).

2. H&Es refers to a “visualization of the tumor via hematoxylin and eosin (H&E) staining. The H&E staining technique allows for characterization of background tumor architecture and individual cell morphology by highlighting nuclei in blue and cytoplasm in red. Recognition of characteristic histologic features allows the pathologist to narrow the diagnostic possibilities” (Kuhn et al. 2021, 31).

3. Guidance from the Royal College of Pathologists and the Institute of Biomedical Science (2015) on “The Retention and Storage of Pathological Records and Archives Pathology” recommends blocks be preserved permanently, histology slides and smears for ten years, and wet tissue for at least four weeks. The latest guidance reflects on the increasing use of genome-wide sequencing technologies.

4. For large-scale genomic medicine projects such as the 100,000 genomes project, tissue is stored using a fresh-frozen method rather than in paraffin blocks. In this section, we focus exclusively on archiving of, and maintaining potential in, paraffin blocks in anticipation of generating a molecular future for patients.

## References

[bibr1-01622439231172571] Adams Vincanne Michelle Murphy Adele Clarke E. . 2009. “Anticipation: Technoscience, Life, Affect, Temporality.” Subjectivity 28 (1): 246–65.

[bibr2-01622439231172571] Bennett Jane . 2010. Vibrant Matter: A Political Ecology of Things. Durham, NC: Duke University Press.

[bibr3-01622439231172571] Bogicevic Ivana Kristoffer Rohrberg S. Estrid Høgdall Mette Svendsen N. . 2020. “The Somatic Mode: Doing Good in Targeted Cancer Therapy.” New Genetics and Society 40 (2): 178–98.

[bibr4-01622439231172571] Borup Mads Nik Brown Kornelia Konrad Harro Van Lente . 2006. “The Sociology of Expectations in Science and Technology.” Technology Analysis & Strategic Management 18 (3-4): 285–98.

[bibr5-01622439231172571] Bourret Pascale Peter Keating Alberto Cambrosio . 2011. “Regulating Diagnosis in Post-genomic Medicine: Re-aligning Clinical Judgment?” Social Science & Medicine 73 (6): 816–24. 21664021 10.1016/j.socscimed.2011.04.022

[bibr6-01622439231172571] Cambrosio Alberto Peter Keating Etienne Vignola-Gagné Sylvain Besle Pascale Bourret . 2018. “Extending Experimentation: Oncology’s Fading Boundary between Research and Care.” New Genetics and Society 37 (3): 207–26.

[bibr7-01622439231172571] Clarke E. Adele Carrie Friese Rachel Washburn . 2016. Situational Analysis in Practice: Mapping Research with Grounded Theory. London, UK: Routledge.

[bibr8-01622439231172571] d’Hoop Ariane . 2021. “On the Potentialities of Spaces of Care: Openness, Enticement, and Variability in a Psychiatric Center.” Science, Technology, & Human Values 46 (3): 577–99.

[bibr9-01622439231172571] Domagala-Kulawik Joana . 2019. “New Frontiers for Molecular Pathology.” Frontiers in Medicine 6: 284. 31867335 10.3389/fmed.2019.00284 PMC6904313

[bibr10-01622439231172571] Doring Martin Jörg Zinken . 2005. “The Cultural Crafting of Embryonic Stem Cells: The Metaphorical Schematisation of Stem Cell Research in the Polish and French Press.” Metaphorik.de 8: 6–33.

[bibr11-01622439231172571] Franklin Sarah . 2006. “Embryonic Economies: The Double Reproductive Value of Stem Cells.” BioSocieties 1 (1): 71–90.

[bibr12-01622439231172571] Friese Carrie . 2013. “Realizing Potential in Translational Medicine: The Uncanny Emergence of Care as Science.” Current Anthropology 54 (S7): S129–38.

[bibr13-01622439231172571] Ganchoff Chris . 2004. “Regenerating Movements: Embryonic Stem Cells and the Politics of Potentiality.” Sociology of Health & Illness 26 (6): 757–74. 15383040 10.1111/j.0141-9889.2004.00417.x

[bibr14-01622439231172571] Hiley T. Crispin John Le Quesne George Santis Rowena Sharpe David De Castro Gonzalez Gary Middleton Charles Swanton . 2016. “Challenges in Molecular Testing in Non-small-cell Lung Cancer Patients with Advanced Disease.” Lancet 388: 1002–11. 27598680 10.1016/S0140-6736(16)31340-X

[bibr16-01622439231172571] Kerr Anne Choon Chekar Key Emily Ross Julia Swallow Sarah Cunningham-Burley . 2021. Personalised Cancer Medicine: Future Crafting in the Genomic Era. Manchester, UK: Manchester University Press. 33555770

[bibr17-01622439231172571] Kitzinger Jenny Clare Williams . 2005. “Forecasting Science Futures: Legitimising Hope and Calming Fears in the Embryo Stem Cell Debate.” Social Science & Medicine 61 (3): 731–40. 15899330 10.1016/j.socscimed.2005.03.018

[bibr18-01622439231172571] Kuhn J. Karin Jeffrey Cloutier M. Robert Boutin D. Robert Steffner Geoffrey Riley . 2021. “Soft Tissue Pathology for the Radiologist: A Tumor Board Primer with 2020 WHO Classification Update.” Skeletal Radiology 50 (1): 29–42. 32743671 10.1007/s00256-020-03567-w

[bibr19-01622439231172571] Landecker Hannah . 2009. Culturing Life. Cambridge, UK: Harvard University Press.

[bibr20-01622439231172571] Lappé Martine Hannah Landecker . 2015. “Sociology in an Age of Genomic Instability: Copy Number Variation, Somatic Mosaicism, and the Fallen Genome.” In Brea Parry L. (ed.) Genetics, Health and Society (Advanced in Medical Sociology, Vol. 16). Bingley, UK: Emerald Group, pp. 157–186.

[bibr21-01622439231172571] Lee Jieun . 2016. “Placental Economies: Care, Anticipation and Vital Matters in the Placenta Stem Cell Lab in Korea.” BioSocieties 11 (4): 458–75.

[bibr22-01622439231172571] Michael Mike . 2017. “Enacting Big Futures, Little Futures: Toward an Ecology of Futures.” The Sociological Review 65 (3): 509–24.

[bibr23-01622439231172571] Mitchell Robert Catherine Waldby . 2006. Tissue Economies: Durham, UK: Duke University Press.

[bibr24-01622439231172571] Parry Sarah . 2006. “(Re)constructing Embryos in Stem Cell Research: Exploring the Meaning of Embryos for People Involved in Fertility Treatments.” Social Science & Medicine 62 (10): 2349–59. 16289740 10.1016/j.socscimed.2005.10.024

[bibr25-01622439231172571] The Royal College of Pathologists and the Institute of Biomedical Science. 2015, April. “The Retention and Storage of Pathological Records and Specimens (5th Edition).” https://www.rcpath.org/resourceLibrary/the-retention-and-storage-of-pathological-records-and-specimens–5th-edition-.html. (Accessed 1 March 2023)

[bibr26-01622439231172571] Star L. Susan . 2010. “This Is Not a Boundary Object: Reflections on the Origin of a Concept.” Science, Technology, & Human Values 35 (5): 601–17.

[bibr27-01622439231172571] Svendsen N. Mette , 2011. “Articulating Potentiality: Notes on the Delineation of the Blank Figure in Human Embryonic Stem Cell Research.” Cultural Anthropology 26 (3): 414–37.

[bibr28-01622439231172571] Svendsen N. Mette Lene Koch . 2013. “Potentializing the Research Piglet in Experimental Neonatal Research.” Current Anthropology 54 (S7): S118–28.

[bibr29-01622439231172571] Swallow Julia Anne Kerr Choon Chekar Key Sarah Cunningham-Burley . 2020. “Accomplishing an Adaptive Clinical Trial for Cancer: Valuation Practices and Care Work across the Laboratory and the Clinic.” Social Science and Medicine 242: 112949. 10.1016/j.socscimed.2020.112949 PMC7193889 32278242

[bibr30-01622439231172571] Taussig Karen-Sue Klaus Hoeyer Stefan Helmreich . 2013. “The Anthropology of Potentiality in Biomedicine: An Introduction to Supplement 7.” Current Anthropology 54 (S7): S3–S14.

[bibr31-01622439231172571] Van Lente Harro Arie Rip . 2012. “Expectations in Technological Developments: An Example of Prospective Structures to be Filled in by Agency.” In Getting New Technologies Together, edited by Disco Cornelis Meulen Barend van der , 203–30. Berlin, Germany: De Gruyter.

[bibr32-01622439231172571] Waldby Catherine . 2019. The Oocyte Economy. Durham, UK: Duke University Press.

[bibr33-01622439231172571] Williams Clare Jenny Kitzinger Lesley Henderson . 2003. “Envisaging the Embryo in Stem Cell Research: Rhetorical Strategies and Media Reporting of the Ethical Debates.” Sociology of Health & Illness 25 (7): 793–814. 19774747 10.1046/j.1467-9566.2003.00370.x

